# Probing Human Telomeric DNA and RNA Topology and Ligand Binding in a Cellular Model by Using Responsive Fluorescent Nucleoside Probes

**DOI:** 10.1002/cbic.201700283

**Published:** 2017-07-10

**Authors:** Sudeshna Manna, Cornelia H. Panse, Vyankat A. Sontakke, Sarangamath Sangamesh, Seergazhi G. Srivatsan

**Affiliations:** ^1^ Department of Chemistry Indian Institute of Science Education and Research (IISER) Dr. Homi Bhabha Road Pune 411008 India

**Keywords:** fluorescent probes, G-quadruplexes, micelles, nucleosides, telomeric repeats

## Abstract

The development of biophysical systems that enable an understanding of the structure and ligand‐binding properties of G‐quadruplex (GQ)‐forming nucleic acid sequences in cells or models that mimic the cellular environment would be highly beneficial in advancing GQ‐directed therapeutic strategies. Herein, the establishment of a biophysical platform to investigate the structure and recognition properties of human telomeric (H‐Telo) DNA and RNA repeats in a cell‐like confined environment by using conformation‐sensitive fluorescent nucleoside probes and a widely used cellular model, bis(2‐ethylhexyl) sodium sulfosuccinate reverse micelles (RMs), is described. The 2′‐deoxy and ribonucleoside probes, composed of a 5‐benzofuran uracil base analogue, faithfully report the aqueous micellar core through changes in their fluorescence properties. The nucleoside probes incorporated into different loops of H‐Telo DNA and RNA oligonucleotide repeats are minimally perturbing and photophysically signal the formation of respective GQ structures in both aqueous buffer and RMs. Furthermore, these sensors enable a direct comparison of the binding affinity of a ligand to H‐Telo DNA and RNA GQ structures in the bulk and confined environment of RMs. These results demonstrate that this combination of a GQ nucleoside probe and easy‐to‐handle RMs could provide new opportunities to study and devise screening‐compatible assays in a cell‐like environment to discover GQ binders of clinical potential.

## Introduction

Guanine‐rich nucleic acid sequences that have the potential to form four‐stranded noncanonical secondary structures, called G‐quadruplexes (GQs), are frequently found in the genome of mammals, bacteria, and viruses.^1^ The position and conservation of putative GQ‐forming motifs, particularly among mammalian species, as evident from bioinformatics and sequencing studies, suggest that GQ could be an important class of structural element for gene regulation.^2^ In the human genome, GQ‐forming sequences are mostly found in the telomeric region (e.g., telomeric DNA and RNA repeats) and in several DNA promoters (e.g., C‐myc, C‐kit) and untranslated regions of mRNA (e.g., NRAS, BCL‐2).^3^, ^4^ Recent biochemical investigations indicate that these G‐rich sequences play crucial roles in cellular processes, such as chromosome maintenance, as well as transcriptional and translational regulation of several protooncogenes, which have been found to be in consensus with the ability of these sequences to form stable GQ structures in vitro.^5^, ^6^ Consequently, stabilization of GQ structures by small‐molecule ligands has emerged as a novel approach to cancer therapeutics.^7^ In this context, several small‐molecule ligands that bind and stabilize GQs are being rigorously evaluated as chemotherapeutic candidates, and have been used as tools to understand the biological role of GQ‐forming sequences.^8^, ^9^

In terms of structure, G‐rich sequences form wide varieties of geometries in vitro, depending on the sequence and ionic environment.^3^, ^9^ These sequences typically form either one or a combination of antiparallel‐, parallel‐, and mixed parallel–antiparallel‐stranded GQ structures. Several methods based on circular dichroism (CD), fluorescence, NMR spectroscopy, and X‐ray crystallography techniques have provided valuable information on the structure and ligand‐binding affinities of GQs in cell‐free systems.^10^ Notably, FRET pair containing oligonucleotides (ONs),^11^ ligands,^12^ metal complexes,10c and fluorescent nucleobase analogues,^13^ which exhibit changes in their fluorescence properties upon folding, have been widely used to probe the formation and recognition properties of GQs. More recently, fluorescent GQ‐specific ligands and antibodies have been used to visualize DNA and RNA GQs in mammalian cells.^14^ Model systems, which closely mimic the physical properties (e.g., polarity and viscosity) and crowded environment of the cell, have also been used to study the structure, stability, and recognition properties of human telomeric (H‐Telo) DNA and RNA repeats.^15^ Typically, in aqueous buffer, the H‐Telo DNA repeat (TTAGGG)_*n*_ forms an antiparallel GQ in Na^+^ ionic conditions and a combination of GQ topologies in the presence of K^+^ ions, in which hybrid‐type mixed parallel–antiparallel‐stranded GQ structures predominate.^16^ However, crowding agents and highly viscous cosolutes, such as poly(ethylene glycol) (PEG), polysaccharides, and deep eutectic solvents in the presence of K^+^ ions, have been shown to stabilize the parallel GQ structure, which exhibits slower folding dynamics, reduced ligand‐binding affinity, and decreased stabilization by ligands compared with those in dilute buffer solutions.^17^, ^18^, ^19^, ^20^ It has been suggested that the dehydrating nature of crowding agents (e.g., PEG) drives the formation of parallel GQ in the presence of K^+^ ions.^21^ Hence, the use of PEG as a molecular crowding agent in the study of GQs may not be appropriate.^22^ Recent NMR spectroscopic analyses of the H‐Telo DNA repeat in live *Xenopus laevis* oocytes and oocytes egg extract support the notion that cellular environment favors conformations that closely resemble those observed in vitro under K^+^ ionic conditions, as opposed to the parallel topology predicted in the presence of synthetic crowding agents.^23^ However, an equivalent telomeric repeat containing RNA, TERRA (UUAGGG)_*n*_, folds into a parallel GQ structure irrespective of the ionic conditions,^24^ and the topology does not change in the presence of PEG (40 %).15b

Despite extensive studies, probing different GQ topologies and their ligand‐binding abilities in a cellular environment has remained a major challenge.^25^, ^26^, ^27^ For example, structural analysis of GQs in the cell requires elaborate assay setups and expensive isotope‐labeled ONs in non‐apoptotic concentrations, which often leads to obscure signals due to the inhomogeneity of cellular samples.^25^ Furthermore, paucity of efficient biophysical probes that can differentiate and quantitatively report ligand binding to different DNA and RNA GQ structures has hampered the discovery of clinically viable GQ binders. Therefore, the development of screening‐compatible biophysical platforms that would enable the easy detection and estimation of ligands binding to different GQ structures in a cellular model will be highly beneficial not only to advance our understanding of GQ structures and recognition in cellular milieu, but could also support approaches to discover efficient GQ binders.

**Figure 1:**
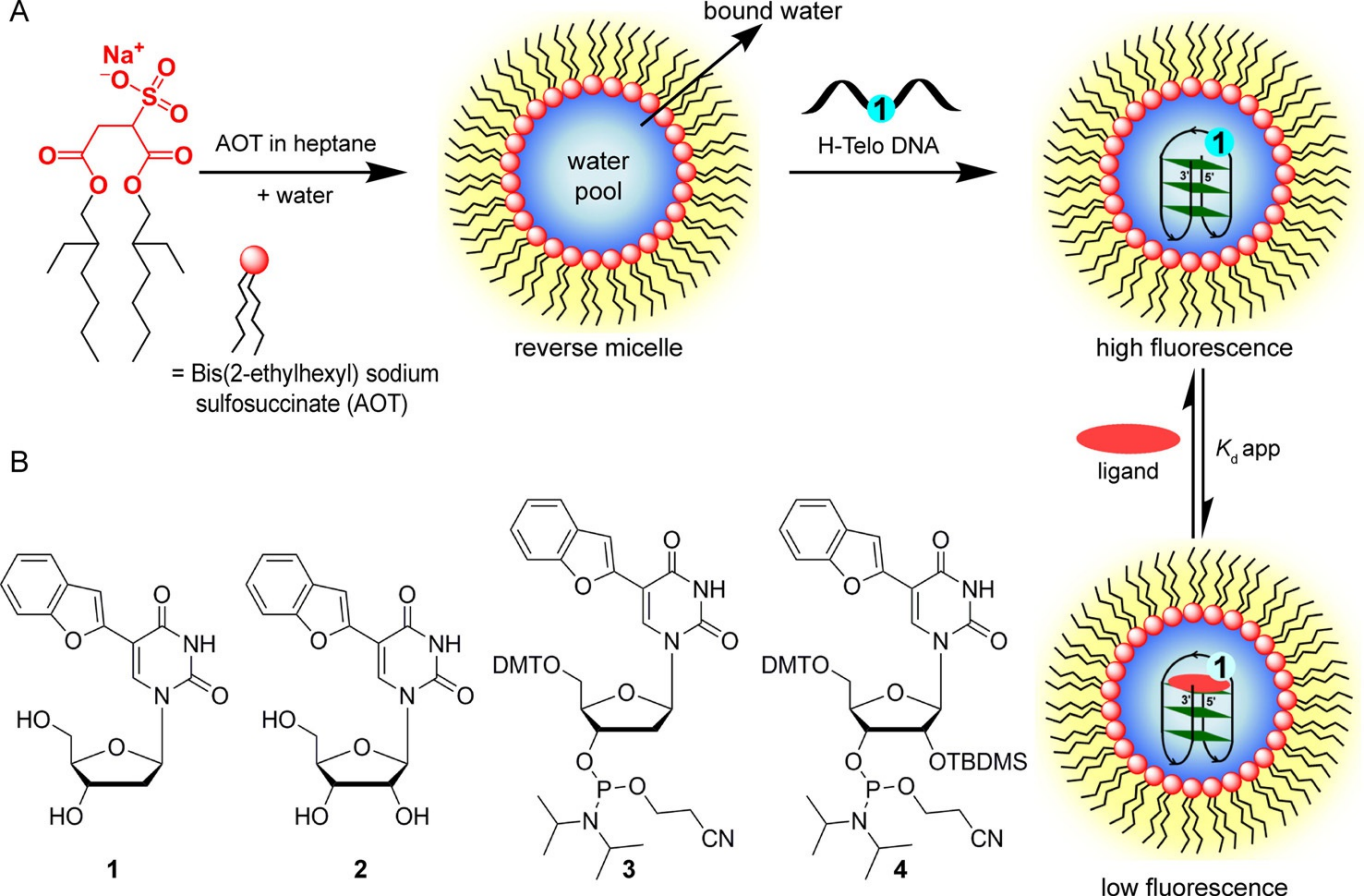
A) A schematic illustration of the platform to study the structure and ligand‐binding ability of GQ‐forming ONs (e.g., H‐Telo DNA repeat) in a cell‐like confined environment by using 5‐benzofuran‐modified 2′‐deoxyuridine (**1**) and bis(2‐ethylhexyl) sodium sulfosuccinate (AOT) RMs. The water pool solubilizes the fluorescently labeled telomeric ON repeats and supports the formation of respective GQ structures. The nucleoside probes photophysically detect the formation of the GQ structure and also help in determining the binding affinity of a ligand to GQ structures in a confined environment. B) Chemical structures of fluorescent 2′‐deoxy (**1**) and ribonucleoside (**2**) analogues and phosphoramidite substrates **3** and **4** used in the synthesis of labeled H‐Telo DNA and RNA ONs, respectively. DMT=4,4′‐dimethoxytrityl, TBDMS=*tert*‐butyldimethylsilyl.

Herein, we describe the development of a platform to investigate the structure and ligand‐binding ability of H‐Telo DNA and TERRA ONs in a cell‐like confined environment by using microenvironment‐sensitive fluorescent nucleoside probes and reverse micelles (RM; Figure 1). The nanosized water pool encapsulated in RMs is an established membrane model, which is known to mimic the physical characteristics and crowded environment of cells.^28^ The emissive 2′‐deoxy and ribonucleoside probes are based on a 5‐benzofuran uracil core,^29^, ^30^ and they faithfully report the environment of water encapsulated in RMs through changes in their fluorescence properties. The useful features of RMs and the conformation sensitivity of the nucleoside probes facilitated a comparison of GQ topologies adopted by H‐Telo DNA and RNA repeats in aqueous buffer and a confined environment. Furthermore, these GQ sensors enabled the development of a simple fluorescence assay to quantify the ligand‐binding ability of H‐Telo DNA and RNA GQ structures in a confined environment. Our results indicate that such emissive GQ sensors could provide new opportunities to study and step‐up discovery assays in a cell‐like environment to identify efficient GQ binders of therapeutic potential.

## Results and Discussion

### Platform design

To establish a fluorescence‐based platform to study GQ structures in a cell‐like confined environment, we chose a combination of a widely used membrane model, AOT RM, and fluorescent GQ sensors, analogues of 5‐benzofuran‐2′‐deoxyuridine (**1**) and 5‐benzofuran uridine (**2**; Figure 1). A ternary mixture of AOT, apolar solvent, and water forms stable micellar aggregates, in which the polar head groups of AOT face the water and hydrophobic chains extend towards the apolar solvent.^31^ The size of the encapsulated water droplet increases linearly with increasing *w*_0_ value (*w*_0_=[water]/[AOT]). Notably, the dynamics, polarity, viscosity, and proton‐transfer efficiency of the aqueous micellar core are quite different from that of bulk water or aqueous buffer, in which most biophysical analyses are performed.^32^ In addition, RMs are transparent and can solubilize biomolecules uniformly; this qualifies them as a convenient model to mimic the confined environment of the cell to study biomolecular structures and dynamics, including nucleic acids, by various spectroscopy techniques.^33^ For example, the effect of confinement on the ON dynamics and conformational flexibility of therapeutically important hairpin RNA motifs, such as HIV TAR and U4 snRNA, has been studied by fluorescence and NMR spectroscopy techniques.^34^, ^35^ In another report, the slower hybridization rate of DNA ONs in RMs has been aptly utilized in detecting the mismatches in DNA duplexes by CD.^36^ More recently, absorption and CD techniques have been used to evaluate the stability and conformation of GQs in RMs formed by anionic and cationic surfactants.^37^, ^38^, ^39^

We have recently introduced fluorescent nucleoside analogues derived by attaching a benzofuran moiety at the 5‐position of **1** and **2**.^29^, ^30^ The nucleoside analogues are reasonably emissive and their fluorescence properties are highly sensitive to changes in solvent polarity and viscosity. Interestingly, upon incorporation into one of the loop residues of the human telomeric DNA and TERRA ONs, the probes photophysically distinguished the formation of different DNA and RNA GQ structures in aqueous buffer.^40^ Together, the environment sensitivity of the nucleoside probes and the ability of the aqueous micellar core to mimic the intracellular environment, as described above, provided the impetus to set up an efficient platform to probe the topology and binding affinity of H‐Telo DNA and RNA repeats in a cell‐like confined environment.

### Benzofuran‐modified nucleoside probe senses the microenvironment of AOT RMs

The water encapsulated in RM is configured into distinct domains, namely, “bound” water, which hydrates the head groups and counterions, and “free” water in the inner core (Figure 1).^41^ Typically at *w*_0_<8, the majority of water molecules interact with head groups and counterions to form a structured domain, which is more viscous and less polar. However, upon increasing *w*_0_ values, a well‐defined water pool emerges, which has higher polarity and lower viscosity than that of water at the interface. In order to evaluate the efficacy of the nucleoside probe to report the microenvironment of AOT RMs, we measured the fluorescence properties of benzofuran‐modified uridine analogues as a function of increasing *w*_0_ values. Although emissive **1** and **2** exhibit similar photophysical properties in solvents of different polarity and viscosity (Table S1 in the Supporting Information),^29^, ^30^ the ribonucleoside analogue (**2**) was preferred in this study because of its higher solubility in AOT RMs.

Nucleoside **2** exhibits a significantly higher quantum yield (0.21) and lifetime (2.55 ns) in water than that in a nonpolar solvent, such as dioxane (0.10 and 0.43 ns, respectively; Table S1). In water, the emission maximum is centered at *λ*≈447 nm, which is considerably blue‐shifted to *λ*=404 nm in dioxane. The presence of a molecular rotor element (rotatable bond between benzofuran and uracil rings) also affected the fluorescence properties of the nucleoside in solvents of different viscosity (Table S1). Increasing the viscosity of the medium from ethylene glycol to glycerol resulted in a discernible increase in fluorescence quantum yield and anisotropy with no apparent change in emission maximum. Collectively, these results indicate that the nucleoside is sensitive to both environment and conformation.^42^ The fluorescence signatures obtained from bulk solvents were further used to determine the responsiveness of the nucleoside analogue in the confined water of AOT RMs.

**Figure 2:**
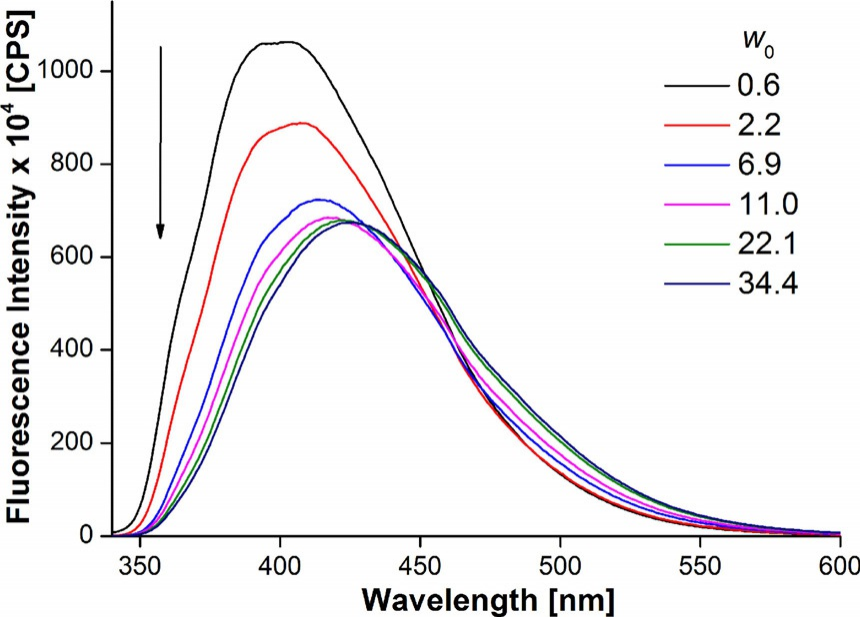
Emission spectra of **2** (1 μm) in AOT RMs (200 mm in *n*‐heptane) at different *w*_0_ values. Samples were excited at *λ*=322 nm with excitation and emission slit widths of 3 and 4 nm, respectively.

RM samples of increasing *w*_0_ value were prepared by adding appropriate amounts of **2** dissolved in water to AOT (sodium salt) in heptane, such that the concentrations of nucleoside and AOT were maintained at 1 μm and 200 mm, respectively. At a low water content (*w*_0_=0.6–2.2), the nucleoside exhibited an emission band at *λ*≈403 nm, which is similar to the *λ*_em_ value in dioxane, but considerably blue‐shifted relative to that in bulk water (*λ*_em_=447 nm; Figure 2, compare Tables 1 and S1). As the water content was increased, discernible quenching in fluorescence intensity accompanied by a red‐shifted emission was observed. The intensity and emission maximum saturated at *w*_0_=11, which apparently is similar to the emission maximum of **2** in methanol (*λ*≈423 nm; Figure 2, Tables 1 and S1). This result is in good agreement with reports in the literature, which have also predicted a polarity equivalent to that of methanol for water encapsulated in AOT RMs.32b, 43 Consistent with the steady‐state fluorescence data, the excited‐state lifetime of the nucleoside decreased with increasing size of the water pool. Similarly, anisotropy measurements revealed a gradual reduction in anisotropy of the nucleoside with increasing *w*_0_ (Table 1). This observation is consistent with the lower viscosity of the aqueous micellar core at higher *w*_0_ than that of the highly viscous water domain at lower *w*_0_.32c

**Table 1:** Fluorescence properties of **2** in AOT RMs (200 mm in *n*‐heptane) as a function of increasing *w*_0_.

*w*_0_	*λ*_em_ [nm]	*τ*_av_^[a]^ [ns]	*r* ^[a]^
0.6	403	1.80	n.d.
2.2	407	1.70	0.192
6.9	414	1.46	0.171
11.0	420	1.48	0.132
22.1	423	1.44	0.108
34.4	427	1.57	n.d.

[a] Standard deviation for average lifetime (*τ*_av_) and anisotropy (*r*) at different *w*_0_ values are ≤0.06 ns and ≤0.006, respectively. n.d.: not determined.

The ability of the nucleoside probe to photophysically report the microenvironment of the water pool in RMs at different *w*_0_ values will depend on the location of the probe within the RM at a given *w*_0_ value. This can be deduced by comparing the fluorescence properties of the nucleoside in bulk solvents and RM. Rigidification of the fluorophore (benzofuran‐modified uracil) in viscous medium will result in an increase in fluorescence intensity and anisotropy, whereas a change in the polarity of the medium from nonpolar to polar will result in an increase in fluorescence intensity and a red shift in the emission maximum. In the absence of a well‐defined water pool at low *w*_0_ values, it is likely that the nucleoside is solubilized by bound water at the AOT–water interface, which is less polar and more viscous (Figure S1).^41^ Hence, we observed an intense blue‐shifted emission band and higher anisotropy due to a nonpolar environment around the rigidified fluorophore (Figure 2 and Table 1). However, if the water/AOT ratio is increased, the probe permeates from a more viscous and nonpolar domain to a less viscous and more polar water pool. Hence, the nucleoside probe located in the aqueous micellar core is derigidified and surrounded by more polar water, resulting in fluorescence quenching, red‐shifted emission band, and reduced anisotropy. From these observations, it can be concluded that the emissive nucleoside analogue faithfully reports the microenvironment of the confined water. Hence, we decided to deploy the nucleoside analogue in a fluorescence assay to probe the structure and recognition properties of H‐Telo DNA and RNA repeats in RM.

### Probing H‐Telo DNA and RNA GQ structures in buffer and AOT RMs

Among the various GQ‐forming sequences, the H‐Telo DNA repeat is the most studied because of its structural polymorphism and biological roles.^2^, ^5^ Telomeric DNA, which end‐caps and protects the chromosomes from degradation and fusion, has been implicated in carcinogenesis and ageing‐related diseases.^6^ Biochemical investigations with GQ‐stabilizing ligands suggest that telomeric DNA uses this structural element to carry out its function. However, it is still not clear which topology it adopts and how different topologies interact with ligands in the cellular environment.

**Figure 3:**
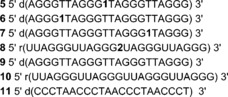
Benzofuran‐modified H‐Telo DNA ONs **5**–**7**: the dT residue in the first (**6**), second (**5**), and third (**7**) loops of H‐Telo DNA ONs was replaced with emissive nucleoside **1**. Benzofuran‐modified TERRA ON **8**: the rU residue in the second loop was replaced with emissive nucleoside **2**. ONs **9** and **10** are control, unmodified H‐Telo DNA and TERRA, respectively. ON **11** is complementary to DNA ONs **5**–**7** and RNA ON **8**.

Telomeric DNA forms various GQ structures in vitro, depending on the ionic conditions (see below).^16^ In general, H‐Telo GQ structures are made of three loops and three G‐tetrads, which are stacked above each other. Notably, the conformation of the loop residues (TTA) in each topology is distinctly different. Hence, we replaced one of the dT residues in the first, second, and third loops of H‐Telo DNA repeat AGGG(TTAGGG)_3_ with **1** and utilized its fluorescence readout to detect and discriminate different GQ structures (Figure 3). The modified H‐Telo DNA ONs **5**–**7** were synthesized by site‐specifically incorporating phosphoramidite **3** by means of the solid‐phase ON synthesis method.

Although TERRA is an integral part of the telomere, the majority of studies have focused on understanding the structural features and function of the telomeric DNA repeat. Biochemical investigations reveal that TERRA plays an important role in the maintenance of the telomere structure, heterochromatinization, and replication.^44^ In this regard, we have synthesized fluorescently modified TERRA ON **8** (U_2_AG_3_)_4_, in which the uridine residue in the middle loop has been replaced with **2** by using corresponding phosphoramidite **4**. Purity and integrity of DNA ONs **5**–**7** and RNA ON **8** were confirmed by HPLC and MALDI‐TOF MS analyses, respectively (Figures S2 and S3, Table S2).

CD and thermal melting studies were performed to investigate the effect of modification on the structure and stability of the GQs of ONs **5**–**10**. ONs **5**–**10** were annealed to form GQ structures in Tris**⋅**HCl buffer containing NaCl or KCl. In a solution of K^+^, DNA ONs **5**–**7** and **9** displayed a positive peak at *λ*≈290 nm and a shoulder at *λ*≈270 nm, which were characteristic of hybrid‐type structures (Figure S4 A).16b In the presence of Na^+^ ions, ONs **5**–**7** and **9** gave a positive peak at *λ*≈ 294 nm and a strong negative peak at *λ*≈263 nm, which was reminiscent of an antiparallel GQ structure (Figure S4 B).16a On the other hand, irrespective of the metal ion (Na^+^ or K^+^), RNA ONs **8** and **10** showed a positive peak at *λ*≈265 nm and a negative peak at *λ*≈240 nm; these are characteristic of a parallel GQ structure (Figure S5).24a, 45 The *T*_m_ values of unmodified and modified DNA and RNA GQs in KCl/NaCl were similar and consistent with those reported in the literature (Figures S6 and S7, Table S3).^46^ These results indicate that benzofuran modification is structurally minimally invasive and does not hamper the formation of respective GQs in the presence of K^+^ and Na^+^ ions.

**Figure 4:**
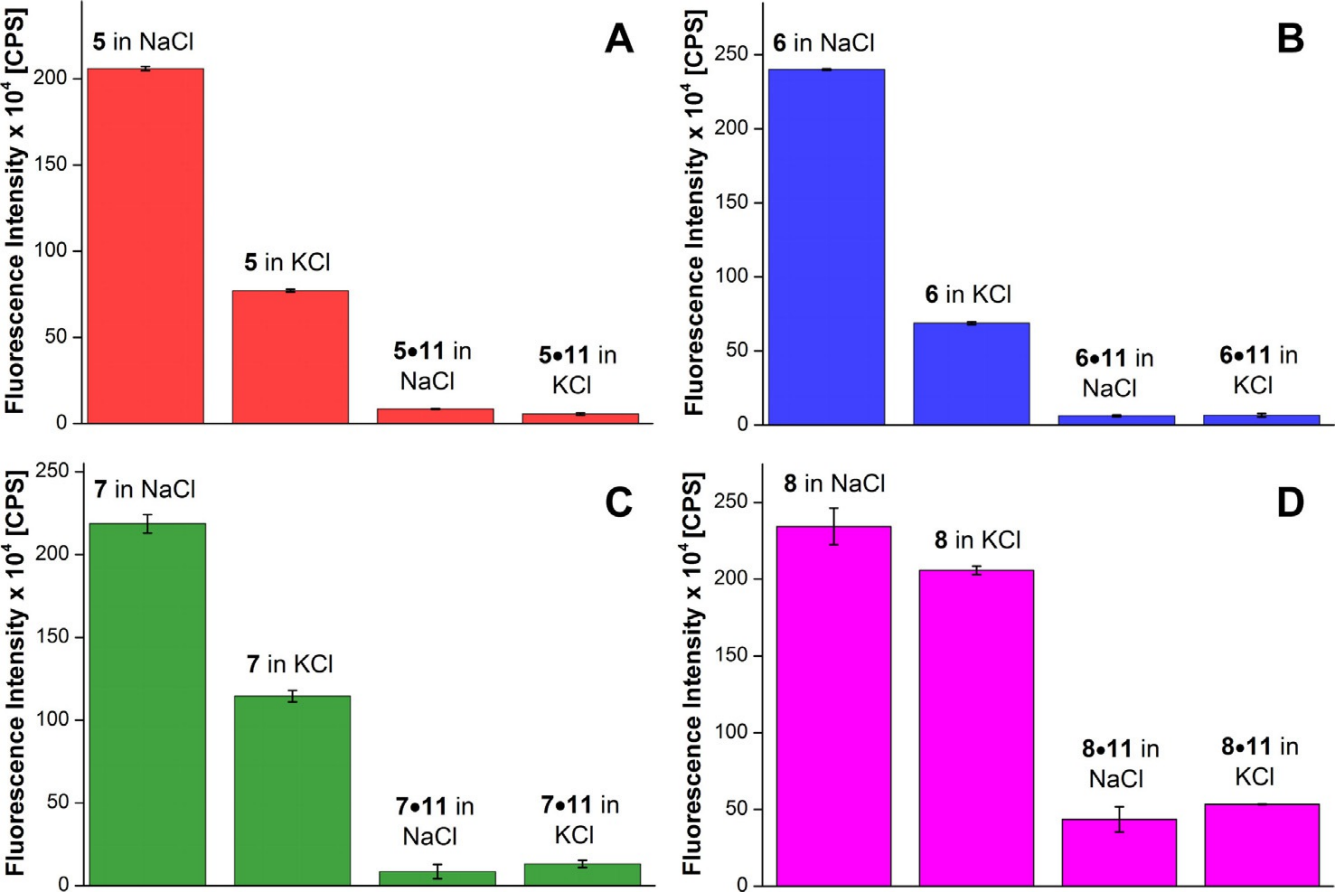
Steady‐state fluorescence spectra of H‐Telo DNA ON A) **5** and corresponding duplex **5⋅11**, B) **6** and corresponding duplex **6⋅11**, C) **7** and corresponding duplex **7⋅11**, and D) TERRA ON **8** and corresponding duplex **8⋅11** in Tris**⋅**HCl buffer buffer (pH 7.5) containing 50 mm NaCl or 50 mm KCl. DNA ON samples (1 μm) were excited at *λ*=322 nm with excitation and emission slit widths of 3 and 4 nm, respectively. RNA ON samples (0.26 μm) were excited at *λ*=322 nm with excitation and emission slit widths of 6 and 8 nm, respectively.

*Detection of H‐Telo DNA GQ structures in aqueous buffer*: DNA ONs **5**–**7** in the presence of K^+^ ions, which predominantly favor the formation of hybrid‐type mixed parallel–antiparallel GQ structures, displayed significant enhancement in fluorescence intensity (nine‐ to 14‐fold), relative to the respective duplexes formed by hybridization with complementary DNA ON **11** (Figure 4 A–C and Figure S8 A–C). Interestingly, under Na^+^ ionic conditions, which are known to induce antiparallel GQ structures, the ONs exhibited further enhancement in fluorescence intensity relative to that of hybrid GQs (two‐ to threefold) and duplexes (17‐ to 40‐fold) under K^+^ and Na^+^ ionic conditions, respectively. The antiparallel structure of ONs **5**–**7** in the presence of Na^+^ ions displayed discernibly higher excited‐state lifetimes than those of hybrid‐type structures formed in the presence of K^+^ ions (Table 2). Furthermore, the minimally perturbing nature of the nucleoside probe, as confirmed by CD and thermal melting analyses, indicates that the observed fluorescence is an outcome of the formation of the respective GQ structures under different ionic conditions.

**Table 2:** Fluorescence properties of GQs of H‐Telo DNA ONs **5**–**7** and respective duplexes in aqueous buffer and AOT RMs at *w*_0_=20.

	ON sample	*λ*_em_ [nm]	*τ*_av_^[a]^ [ns]	ON sample	*λ*_em_ [nm]	*τ*_av_^[a]^ [ns]	ON sample	*λ*_em_ [nm]	*τ*_av_^[a]^ [ns]
aqueous	**4** in NaCl	430	2.07	**5** in NaCl	442	1.65	**6** in NaCl	437	1.78
buffer	**4** in KCl	436	0.72	**5** in KCl	436	1.15	**6** in KCl	435	1.41
	**4⋅7** in NaCl	427	n.d.	**5⋅7** in NaCl	430	n.d.	**6⋅7** in NaCl	433	n.d.
	**4⋅7** in KCl	436	n.d.	**5⋅7** in KCl	430	n.d.	**6⋅7** in KCl	432	n.d.
AOT	**4** in NaCl	430	2.13	**5** in NaCl	434	1.67	**6** in NaCl	432	1.86
RMs	**4** in KCl	430	2.15	**5** in KCl	435	1.68	**6** in KCl	434	1.85
	**4⋅7** in NaCl	427	n.d.	**5⋅7** in NaCl	435	n.d.	**6⋅7** in NaCl	441	n.d.
	**4⋅7** in KCl	429	n.d.	**5⋅7** in KCl	436	n.d.	**6⋅7** in KCl	433	n.d.

[a] The standard deviation for lifetime (*τ*_av_) is ≤0.09. n.d.: not determined. Excited‐state lifetime of duplexes could not be determined because they displayed very low fluorescence.

*Detection of GQ structures of TERRA in aqueous buffer*: RNA ON **8**, in the presence of solutions of Na^+^ and K^+^, showed a highly intense fluorescence band (≈fourfold), relative to that of its duplex **8⋅11** (Figures 4 D and S8 D). Furthermore, through time‐resolved fluorescence analysis, the GQ structure of **8** showed lifetimes of 2.8 and 2.9 ns in the presence of Na^+^ and K^+^ ions, respectively (Table 3). Comparable fluorescence properties exhibited by the GQs of **8** under both Na^+^ and K^+^ ionic conditions are consistent with the parallel GQ structure confirmed by CD analysis (Figure S5).

**Table 3:** Fluorescence properties of GQs of TERRA ON **8** and corresponding duplex **8⋅11** in aqueous buffer and AOT RMs at *w*_0_=20.

	ON sample	*λ*_em_ [nm]	*τ*_av_^[a]^ [ns]
aqueous buffer	**8** in NaCl	441	2.83
	**8** in KCl	443	2.94
	**8⋅11** in NaCl	438	n.d.
	**8⋅11** in KCl	441	n.d.
AOT RMs	**8** in NaCl	435	2.27
	**8** in KCl	432	2.29
	**8⋅11** in NaCl	436	n.d.
	**8⋅11** in KCl	436	n.d.

[a] The standard deviation for lifetime (*τ*_av_) is ≤0.07. n.d.: not determined. Excited‐state lifetime of the duplex in NaCl and KCl could not be determined because it displayed very low fluorescence.

**Figure 5:**
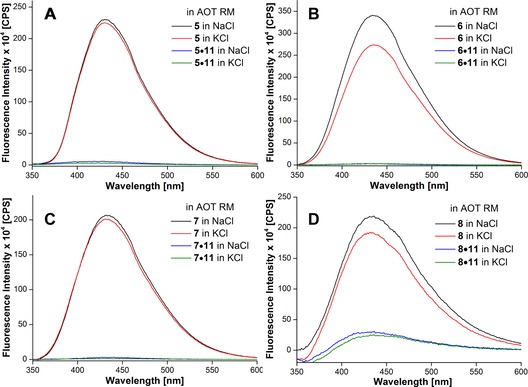
Steady‐state fluorescence spectra of H‐Telo DNA ON A) **5** and corresponding duplex **5⋅11**, B) **6** and corresponding duplex **6⋅11**, C) **7** and corresponding duplex **7⋅11**, and D) TERRA ON **8** and corresponding duplex **8⋅11** in AOT RMs (200 mm in *n*‐heptane) at *w*_0_=20 containing 50 mm NaCl or 50 mm KCl. DNA ON samples (1 μm) were excited at *λ*=322 nm with excitation and emission slit widths of 3 and 4 nm, respectively. TERRA samples (0.26 μm) were excited at *λ*=322 nm with excitation and emission slit widths of 6 and 8 nm, respectively.

*Detection of H‐Telo DNA GQ structures in AOT RMs*: The water pool of AOT RMs at *w*_0_≈20 has been suggested to mimic the compartmentalized environment of the cell, and hence, has been used to study conformation dynamics and hybridization rates of ONs by NMR and absorption spectroscopy techniques.^33^, ^34^, ^35^, ^36^, ^37^ An appropriate amount of preformed stock solution of GQs and duplexes prepared from H‐Telo DNA ONs **5**–**7** in Tris**⋅**HCl buffer, containing NaCl or KCl, was added to AOT RMs such that the concentrations of AOT and ON and the value of *w*_0_ were fixed at 200 mm, 1.0 μm, and 20, respectively. Under these conditions, the RM dispersion was stable and completely transparent for several hours. Upon excitation, GQ‐forming ONs **5**–**7** displayed very high fluorescence intensity, whereas the corresponding duplexes were showed very weak fluorescence (Figure 5). Unlike in aqueous buffer, in which the ONs showed noticeable differences in their fluorescence intensity under Na^+^ and K^+^ ionic conditions, they exhibited similar fluorescence profiles in AOT RMs, irrespective of added salt (Figure 5). The excited‐state lifetimes of H‐Telo DNA ONs in RMs containing NaCl or KCl were also similar and matched well with the lifetime of the antiparallel GQ structure formed in aqueous buffer containing NaCl (Table 2). These results suggest that ONs **5**–**7** in RMs adopt antiparallel GQ structures, irrespective of the type of added monovalent cations. This notion was further supported by CD experiments, wherein the GQ‐forming ONs produced similar CD profiles in AOT RMs containing either Na^+^ or K^+^ ions that were characteristic of an antiparallel GQ structure (Figure S9).16a

Similar to other studies, we have also used the readily available sodium salt of AOT (200 mm) to form stable RMs. The conversion of hybrid structures to an antiparallel GQ structure in RM is possibly due to the exchange of K^+^ ions of the ONs with Na^+^ ions of the AOT head group. This structural conversion would depend on the ratio of Na^+^ to K^+^ ions. Such interconversion of telomeric GQ structures as a function of different Na^+^ to K^+^ ratios has been analyzed by CD experiments.16b, 47 In order to ascertain the structural transformation happening in the RM core, we performed fluorescence and CD studies by using H‐Telo DNA ON **5** in an aqueous buffer containing different ratios of Na^+^/K^+^ ions. The hybrid form of ON **5** in the presence of K^+^ ions gave a reasonably intense fluorescence band as before; this did not change upon increasing the amount of Na^+^ ions up to a ratio of 1:1 (Figure S10 A). Further addition of Na^+^ ions (Na^+^/K^+^=4:1) resulted in a significant increase in fluorescence intensity, as a result of the conversion of hybrid to antiparallel structure. Similar results were obtained from CD experiments (Figure S10 B). In the experiments performed with RMs, the concentration of Na^+^/K^+^ was maintained at 4:1, and hence, ON **5** would have adopted an antiparallel GQ structure. Collectively, these results are consistent with reports in the literature that the interconversion of telomeric GQ structures of similar sequences relies on Na^+^ and K^+^ ion exchange.16b, 47

Attempts to induce the formation GQ structures supported by K^+^ ions by increasing the KCl concentration or by using the potassium salt of AOT failed because these conditions collapsed the RMs. Nevertheless, the emissive nucleoside, irrespective of the position of modification, effectively signaled the formation of GQ structure with enhancement in fluorescence intensity in aqueous buffer and in a confined environment. This feature of the nucleoside probe is advantageous because most fluorophores, when placed in the vicinity of a guanine residue, exhibit drastically quenched emission, which hampers their practical application.^48^

*Detection of GQ structures of TERRA in AOT RMs*: Samples were prepared by adding appropriate amounts of preformed stock solution of TERRA ON **8** and corresponding duplex (**8⋅11**) in Tris**⋅**HCl buffer, containing NaCl or KCl, to AOT RMs such that the concentrations of AOT and ON and the value of *w*_0_ were fixed at 200 mm, 0.26 μm, and 20, respectively. Upon excitation, TERRA **8** displayed an intense fluorescence band in AOT, which was similar under both NaCl and KCl ionic conditions (Figure 5 D). The fluorescence intensity of the GQ of **8** was significantly higher than that of the corresponding duplex **8⋅11** in AOT. Furthermore, the excited‐state decay kinetics of **8** in RMs containing NaCl or KCl revealed similar lifetimes of about 2.3 ns (Table 3). These results suggest that, similar to experiments in aqueous buffer, irrespective of the ionic conditions, TERRA ON **8** adopts a parallel structure in AOT RMs. This observation was confirmed by recording the CD profile of **8** and control, unmodified TERRA ON **10** in AOT RMs containing either 50 mm NaCl or 50 mm KCl. Consistent with earlier reports,24a, 45 the CD profiles of both modified and unmodified TERRA ONs showed a distinct positive peak at *λ*=265 nm and a negative peak at *λ*=240 nm; these were characteristic of a parallel GQ structure (Figure S11).

### Probing ligand binding in aqueous buffer and RMs

**Figure 6:**
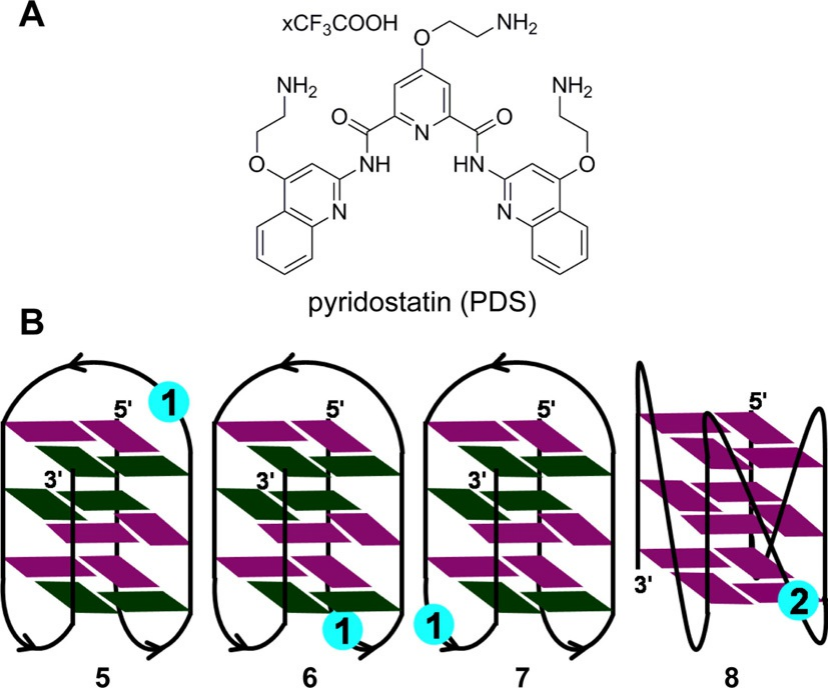
A) Chemical structure of the GQ binder, PDS, used herein. B) The position of nucleoside **1** in different loops of the antiparallel GQ structure of ONs **5**–**7** is shown. In ON **5**, nucleoside **1** is placed in the diagonal loop. In ONs **6** and **7**, nucleoside **1** is placed in the lateral loops. The position of ribonucleoside **2** in the propeller loop of the parallel GQ structure of RNA ON **8** is shown. The *syn* and *anti* guanosines are colored in green and purple, respectively.

**Figure 7:**
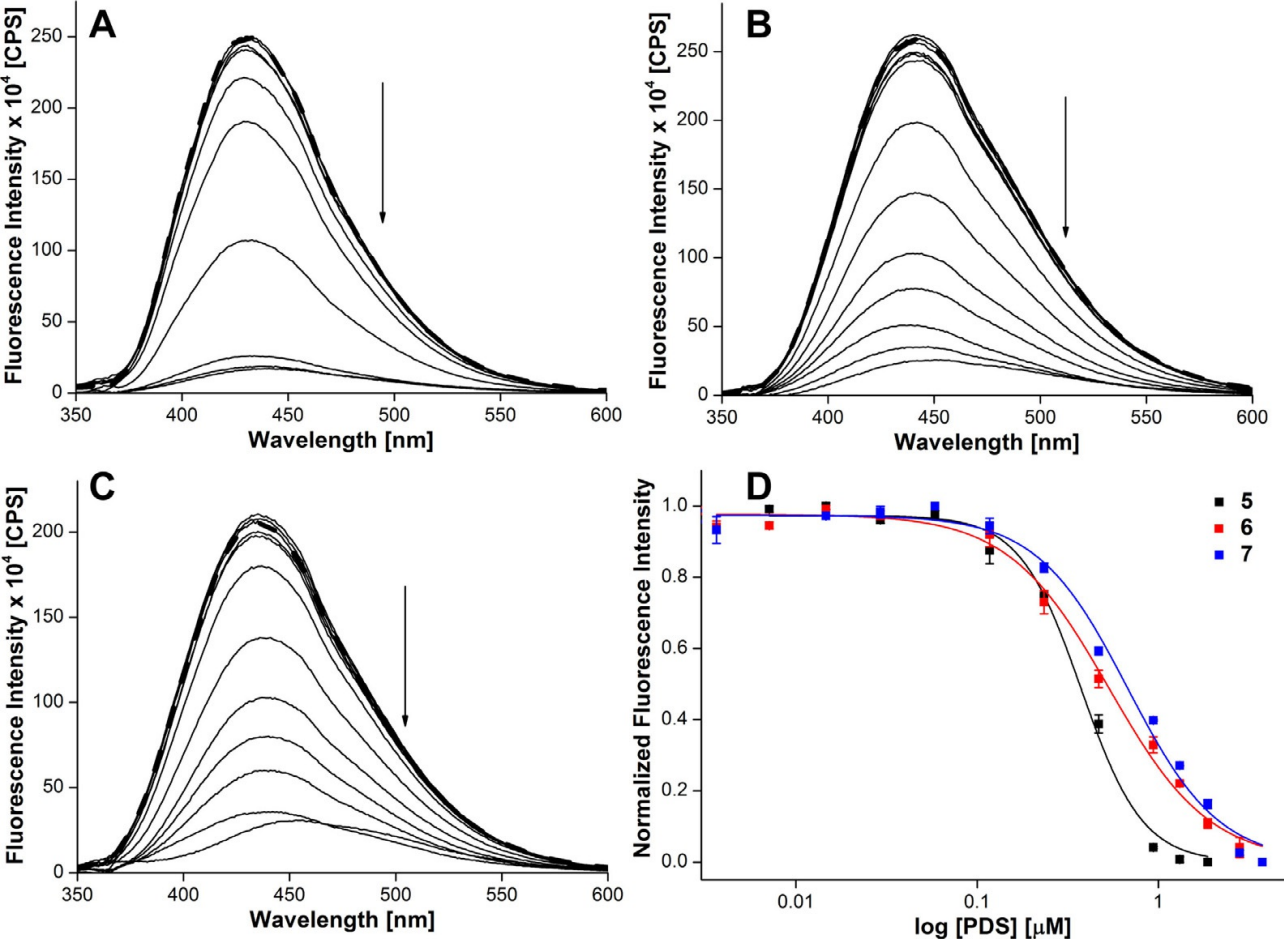
Emission spectra of H‐Telo DNA ONs A) **5**, B) **6**, and C) **7** in aqueous buffer (pH 7.5) containing NaCl (50 mm) as a function of increasing concentration of PDS. The dashed line represents the fluorescence spectrum of GQ ONs in the absence of PDS. The ON (0.28 μm) samples were excited at *λ*=322 nm with excitation and emission slit widths of 4 and 6 nm, respectively. D) Curve fitting for the binding of PDS to H‐Telo DNA ONs **5**–**7** in aqueous buffer containing NaCl (50 mm). Normalized fluorescence intensity at the respective emission maximum (Table 2) is plotted against log [PDS].

Biochemical and structural investigations document that ligands bind individual domains of a GQ structure with different affinities and induce significant conformational changes, particularly in the loop residues.^49^, ^50^ A simple fluorescence binding assay was designed in aqueous buffer and AOT RMs to evaluate the ability of the nucleoside analogues to signal ligand‐induced conformational changes in the H‐Telo DNA and TERRA GQ structures. DNA and RNA ONs **5**–**8** were titrated with pyridostatin (PDS); a known ligand that binds and alters the function of certain GQ‐forming clusters of human genomic loci, including telomeres and proto‐oncogenes (Figure 6 A).^51^ In order to draw a comparison between bulk solution and RM binding data, ONs were annealed in the presence of NaCl, which would induce the formation of an antiparallel GQ for H‐Telo DNA ONs and a parallel GQ for TERRA ON in both aqueous buffer and RMs (Figure 6 B). Upon addition of PDS to ON **5** containing a modification in the diagonal loop, a dose‐dependent quenching in fluorescence intensity (≈15‐fold), corresponding to an apparent *K*_d_=(0.38±0.02) μm, was obtained in aqueous buffer (Figures 6 B and 7 A). Whereas ONs **6** and **7**, containing modifications in the lateral loops, signaled the binding of PDS with significant quenching in fluorescence intensity, the apparent *K*_d_ values ((0.53±0.02) and (0.68±0.002) μm, respectively) were higher than that of PDS binding to ON **5** (Figures 6 B, 7 C, and 7 D). TERRA ON **8**, in which the modification is in the propeller loop, showed about a fivefold quenching in fluorescence intensity upon increasing the PDS concentration (*K*_d_=(0.36±0.02) μm; Figure 6 B and Figure S12). The differential binding affinity exhibited by PDS is likely to be due to differences in the physicochemical environment of the G‐tetrad near the diagonal, lateral, and propeller loops.^50^ In the absence of a change in emission maximum, fluorescence quenching upon ligand binding could be due to derigidification of the fluorophore or a proximal effect, wherein the close vicinity of a polyaromatic ligand with the fluorophore could induce nonradiative dissipation of energy.^50^, ^52^

**Figure 8:**
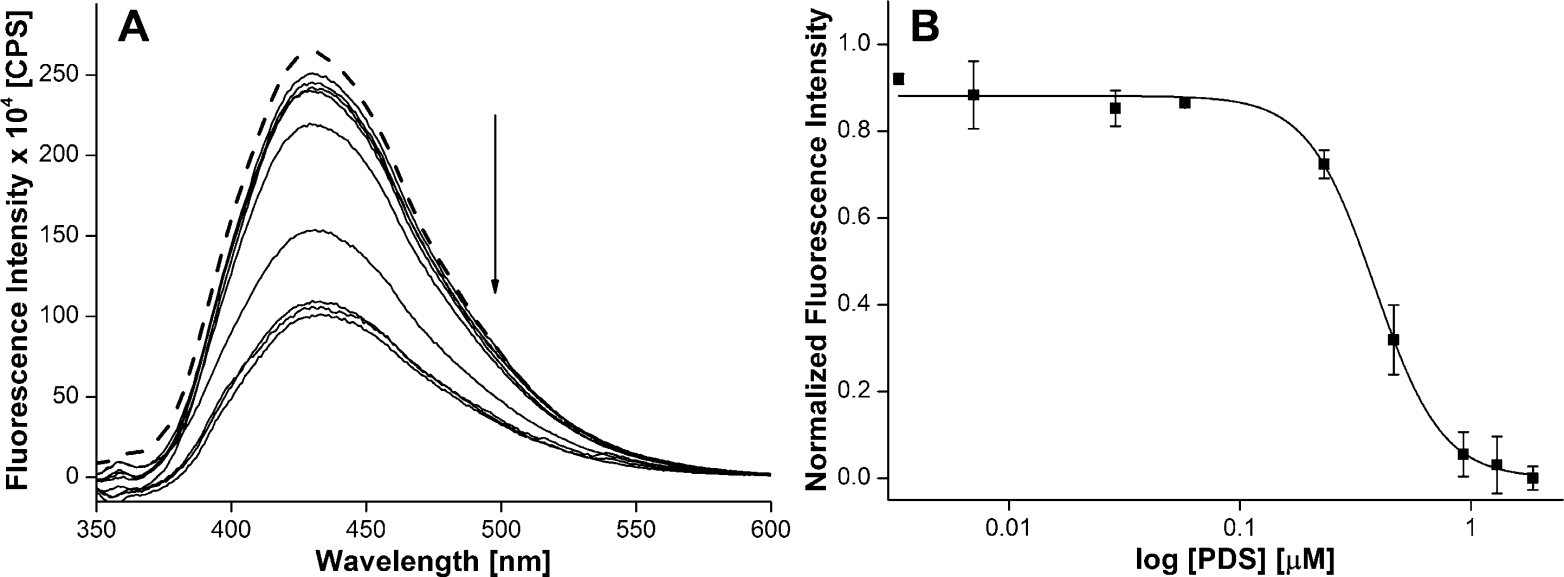
A) Emission spectra of H‐Telo DNA ON **5** (0.28 μm) in AOT RMs containing NaCl (50 mm) as a function of increasing concentration of PDS. The dashed line represents the fluorescence spectrum of **5** in the absence of PDS. The samples were excited at *λ*=322 nm with excitation and emission slit widths of 4 and 6 nm, respectively. B) Curve fitting for the binding of PDS to H‐Telo DNA ON **5** in AOT RMs. Normalized fluorescence intensity at *λ*=430 nm is plotted against log [PDS].

**Figure 9:**
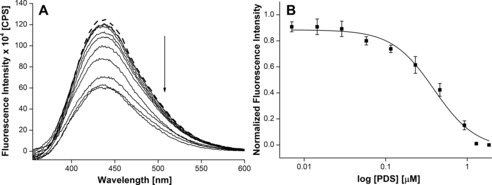
A) Emission spectra of TERRA ON **8** (0.26 μm) in AOT RMs containing NaCl (50 mm) as a function of increasing concentration of PDS. The dashed line represents the fluorescence spectrum of **8** in the absence of PDS. The samples were excited at *λ*=322 nm with excitation and emission slit widths of 6 and 8 nm, respectively. B) Curve fitting for the binding of PDS to TERRA ON **8** in AOT RMs. Normalized fluorescence intensity at *λ*=435 nm is plotted against log [PDS].

The applicability of nucleoside probes **1** and **2** in estimating the binding of ligand to H‐Telo DNA and TERRA GQs in confined environments was studied by preparing stock solutions of DNA and RNA ONs **5**–**8** containing increasing concentrations of PDS. The individual stock solution was then added to AOT RMs, such that *w*_0_ was maintained at 20. The 2′‐deoxyuridine analogue **1**, placed in the diagonal loop of the antiparallel GQ structure of ON **5**, showed significant quenching (≈threefold) in fluorescence intensity as a function of increasing PDS concentration (Figure 8). The extent of quenching observed in RMs was less than that in aqueous buffer, which could be due to the more viscous nature of the water pool compared with that of bulk water.32c Nevertheless, the dose‐dependent quenching in fluorescence intensity enabled the determination of *K*_d_ in RMs ((0.38±0.05) μm), which was very close to that of the *K*_d_ in aqueous buffer. DNA ONs **6** and **7**, wherein the modification is in the lateral loops, exhibited only minor changes in fluorescence intensity upon increasing the ligand concentration, which did not yield reliable *K*_d_ values (Figure S13). This observation is interesting because the relatively stronger binding event, that is, PDS binding to the tetrad near the diagonal loop, is not affected by the cell‐like confined environment, whereas the binding of PDS to the tetrad near the lateral loop, which is weaker, is affected by confinement. TERRA ON **8**, in which the modification is in the propeller loop, showed noticeable quenching (≈twofold) in fluorescence intensity in a dose‐dependent manner upon addition of PDS, which gave a *K*_d_ value of (0.39±0.05) μm (Figure 9). This *K*_d_ value is close to that observed in aqueous buffer. Taken together, these results reveal that compartmentalization and the chemical environment of different domains of the GQ structure determine the binding preference and affinity of ligands to DNA and RNA GQs.

## Conclusion

The conformation sensitivity and ability of benzofuran‐modified fluorescent nucleoside analogues **1** and **2** to sense the microenvironment of confined water of AOT RMs have been aptly utilized in investigating the GQ structures of H‐Telo DNA and RNA ONs in a synthetic cellular model by means of fluorescence spectroscopy. These novel GQ sensors have essentially facilitated a direct comparison of the structure and ligand‐binding affinity of H‐Telo DNA and RNA ON repeats in aqueous buffer and in the cell‐like confined environment of RMs. The present platform, based on these nucleoside probes and RMs, could be highly suitable for setting up screening formats that mimic cellular environment to discover efficient GQ binders with therapeutic potential. In particular, an investigation of the structure and ligand‐binding ability of G‐rich sequences, which form similar GQ topologies, irrespective of the ionic conditions (e.g., TERRA, C‐myc, NRAS) will greatly benefit from this platform.

## Experimental Section

**Fluorescence study of nucleoside 2 in AOT RMs**: To study the fluorescence properties of nucleoside **2** in AOT RMs as a function of increasing *w*_0_ values, samples were prepared by adding appropriate volumes of **2** in water to AOT in *n*‐heptane such that the final ON and AOT concentrations were maintained at 1 μm and 200 mm, respectively. Samples were sonicated for 30 s and equilibrated at room temperature for 3 h before use. For steady‐state experiments, samples were excited at *λ*=322 nm with excitation and emission slit widths of 3 and 4 nm, respectively. All measurements were performed in triplicate in a microfluorescence cuvette (Hellma, path length 1.0 cm) at 20 °C. To study excited‐state decay kinetics of nucleoside **2** in AOT RMs at different *w*_0_ values, samples (1 μm) were excited by using a *λ*=339 nm diode laser source (IBH, UK, NanoLED‐339L) and the emission signal was collected at the respective emission maximum. All experiments were performed in triplicate and lifetimes were calculated by fitting the decay profiles in IBH DAS6 software. The *χ* ^2^ value for all curve fits was found to be nearly unity. Steady‐state anisotropy measurements were performed by exciting the samples at *λ*=322 nm. The anisotropy value (*r*) was determined by analyzing the data by using software provided with the instrument. Anisotropy measurements were performed in triplicate and the values reported herein were an average of 10 successive measurements for each sample.

**Solid‐phase synthesis of modified DNA and RNA ONs**: Benzofuran‐modified H‐Telo DNA ONs **5**–**7** were synthesized on a 1.0 μmol scale (1000 Å controlled pore glass (CPG) solid support) by means of a standard DNA ON synthesis protocol with phosphoramidite **3**.^29^ After the final detritylation step, the solid support was treated with a 30 % aqueous solution of ammonium hydroxide for 24 h at 55 °C. The solution was evaporated to dryness by using a SpeedVac. Benzofuran‐modified TERRA ON **8** was synthesized on a 1.0 μmol scale (1000 Å CPG solid support) by means of a standard RNA ON synthesis protocol with phosphoramidite **4**.^40^ After trityl deprotection on the synthesizer, the solid support was treated with a 1:1 solution (1.5 mL) of methylamine (10 m) in ethanol and water for 12 h. The mixture was then centrifuged, and the supernatant was evaporated to dryness on a SpeedVac. The residue was dissolved in DMSO (100 μL) and TEA**⋅**3 HF (150 μL; TEA=triethylamine) was added. The resulting solution was heated at 65 °C for 2.5 h and slowly cooled to RT. The solution of deprotected RNA ON was lyophilized to dryness. The DNA and RNA ON residues were purified by 20 % PAGE under denaturing conditions. The band corresponding to the full‐length modified ON product was identified by means of UV shadowing, and cut and transferred to a Poly‐Prep column (Bio‐Rad). The gel pieces were crushed with a sterile glass rod, and the ON was extracted by using ammonium acetate buffer (0.5 m, 3 mL) for 12 h and desalted with Sep‐Pak classic C_18_ cartridges (Waters). The purity of ONs **5**–**8** was examined by reversed‐phase (RP) HPLC and characterized by MALDI TOF MS analyses (Figures S2 and S3, Table S2).

**Steady‐state and time‐resolved fluorescence studies of H‐Telo DNA and RNA ONs in buffer and AOT RMs**

*Sample preparation in aqueous buffer*: Fluorescent H‐Telo DNA ONs **5**–**7** (1 μm) and RNA ON **8** (0.26 μm) were annealed at 90 °C for 3 min in Tris**⋅**HCl buffer (10 mm, pH 7.5) containing either NaCl or KCl (50 mm). The samples were slowly cooled to RT and kept at about 4 °C overnight. DNA (1 μm) and RNA (0.26 μm) duplexes were formed by heating a 1:1 mixture of H‐Telo DNA or RNA ONs and complementary ON **11** at 90 °C for 3 min in Tris**⋅**HCl buffer (10 mm, pH 7.5) containing either NaCl or KCl (50 mm). The solutions were slowly cooled to RT and were kept at about 4 °C overnight.

*Sample preparation in RMs*: Stock solutions of H‐Telo DNA ONs and their corresponding duplexes were prepared in Tris**⋅**HCl buffer (10 mm, pH 7.5) containing either NaCl or KCl (50 mm), as mentioned above. An appropriate amount of the solution of annealed DNA ON was mixed with AOT in *n*‐heptane RMs such that the final concentrations of DNA ON and AOT and the value of *w*_0_ were 1 μm, 200 mm, and 20, respectively. The final volume of the DNA sample was 486 μL. The sample of RNA ON was prepared similarly by maintaining final concentrations of RNA ON and AOT and the value of *w*_0_ of 0.26 μm, 200 mm, and 20, respectively. The final volume of RNA sample was 243 μL. The samples were sonicated for 30 s and equilibrated at RT for 3 h.

*Fluorescence analysis*: Steady‐state fluorescence measurements of DNA ON samples in aqueous buffer and AOT RMs were performed by exciting the samples at *λ*=322 nm with excitation and emission slit widths of 3 and 4 nm, respectively. Steady‐state fluorescence measurements of RNA ON samples in aqueous buffer and AOT RMs were performed by exciting the samples at *λ*=322 nm with excitation and emission slit widths of 6 and 8 nm, respectively. An excited‐state decay kinetics study of ONs was performed by exciting the samples with a *λ*=339 nm diode laser source (IBH, UK, NanoLED‐339L). The fluorescence signal was collected at the respective emission maximum and analyzed by using IBH DAS6 analysis software. Lifetime measurements of DNA ON samples were performed in triplicate and lifetime measurements of RNA ONs samples were performed in duplicate. The *χ* ^2^ values for all curve fits were close to unity.

**CD measurements**: ON samples were annealed and prepared for CD analysis in aqueous buffer and AOT RMs as above. The CD spectra of fluorescently modified H‐Telo DNA and RNA ONs **5**–**8** (8 μm) and control unmodified H‐Telo DNA and RNA ONs **9** and **10** (8 μm) in Tris**⋅**HCl buffer (10 mm, pH 7.5) and in AOT RMs containing either NaCl or KCl (50 mm) were recorded from *λ*=200 to 350 nm on a J‐815 CD spectropolarimeter (Jasco, USA) by using a 1 nm bandwidth at 20 °C. Each CD profile is an average of three scans collected at a scan speed of 100 nm min^−1^. CD measurements were performed in duplicate and all spectra were corrected by using an appropriate blank solution in the absence of ONs.

**Thermal melting analysis**: Fluorescently modified H‐Telo DNA and RNA ONs **5**–**8** (1 μm), and control unmodified H‐Telo DNA and RNA ONs **9** and **10** (1 μm), were annealed by heating at 90 °C for 3 min in 10 mm Tris**⋅**HCl buffer (pH 7.5) containing either KCl or NaCl (50 mm). The samples were cooled to RT and kept in an ice bath for at least 1 h. Thermal melting analysis was performed by using a Cary 300Bio UV/Vis spectrophotometer. The temperature was increased from 20 to 90 °C at 1 °C min^−1^ and the absorbance was measured every 1 °C interval at *λ*=295 nm. Forward and reverse cycles were used to determine the *T*_m_ values.

**Fluorescence binding assay: PDS binding to H‐Telo DNA and RNA ONs 5–8 in aqueous buffer and AOT RMs**: The GQ structures of **5**–**8** were formed by annealing the ONs in Tris**⋅**HCl buffer (pH 7.5) containing 50 mm NaCl at 90 °C for 3 min. The samples were cooled to RT and kept at about 4 °C for overnight.

*Binding studies in aqueous buffer*: A series of DNA ON samples (0.28 μm) in Tris**⋅**HCl buffer (10 mm, 50 mm NaCl) containing increasing concentrations of PDS (4 nm to 4 μm) were prepared and incubated at RT for 30 min. Samples were excited at *λ*=322 nm with excitation and emission slit widths of 4 and 6 nm, respectively. Similarly, RNA ON samples (0.26 μm) in Tris**⋅**HCl buffer (10 mm, 50 mm NaCl) containing increasing concentration of PDS (4 nm to 3 μm) were prepared and incubated at RT for 30 min. Samples were excited at *λ*=322 nm with excitation and emission slit widths of 6 and 8 nm, respectively. Fluorescence experiments were performed in duplicate in a microfluorescence cuvette at 20 °C. An appropriate blank in the absence of ONs, but containing the respective concentration of ligand, was subtracted from the individual spectrum.

*Binding studies in AOT RMs*: A series of stock solutions of H‐Telo DNA ONs (3.8 μm) were prepared in Tris**⋅**HCl buffer (pH 7.5, 50 mm NaCl) containing increasing concentrations of PDS. The samples were incubated at RT for 30 min. An individual DNA‐PDS stock solution (36 μL) was added to AOT RMs (450 μL) such that the final concentrations of the ON and AOT and the value of *w*_0_ were 0.28 μm, 200 mm, and 20, respectively. The final volume of the sample was 486 μL, and the concentration range of PDS was between 4 nm and 4 μm. Similarly, stock solutions of H‐Telo RNA ON **8** (3.5 μm) were prepared in Tris**⋅**HCl buffer (pH 7.5, 50 mm NaCl) containing increasing concentrations of PDS. The samples were incubated at RT for 30 min. An individual RNA‐PDS stock solution (18 μL) was added to AOT RMs (225 μL) such that the final concentrations of ON and AOT and the value of *w*_0_ were 0.26 μm, 200 mm, and 20, respectively. The final volume of the sample was 243 μL and the concentration range of PDS was between 4 nm and 3 μm. All samples were sonicated for 30 s and equilibrated at RT for 3 h. Fluorescence spectra were recorded as described above.

From the dose‐dependent quenching curves, the apparent dissociation constants (*K*_d_) for the binding of PDS to H‐Telo DNA and RNA ONs **5**–**8** in aqueous buffer and AOT RMs were determined by fitting a plot of normalized fluorescence intensity (*F*_N_) versus log [PDS] to the Hill equation [Eqs. (1), (2); Origin 8.5].^40^, ^53^

(1) $$F{}_{N}=\frac{F{}_{i}-F{}_{s}}{F{}_{0}-F{}_{s}}$$

*F*_i_ is the fluorescence intensity at each titration point; *F*_0_ and *F*_s_ are the fluorescence intensities in the absence of ligand (L) and at saturation, respectively; and *n* is the Hill coefficient or degree of cooperativity associated with binding.

(2) $$F{}_{N}=F{}_{0}+\left(F{}_{s}-F{}_{0}\right)\left(\frac{\left[L\right]{}^{n}}{\left[K{}_{d}\right]{}^{n}+\left[L\right]{}^{n}}\right)$$

## Conflict of interest

*The authors declare no conflict of interest*.

## Supporting information

As a service to our authors and readers, this journal provides supporting information supplied by the authors. Such materials are peer reviewed and may be re‐organized for online delivery, but are not copy‐edited or typeset. Technical support issues arising from supporting information (other than missing files) should be addressed to the authors.

Supplementary
