# Supplementary material for: Probing Human Telomeric DNA and RNA Topology and Ligand Binding in a Cellular Model by Using Responsive Fluorescent Nucleoside Probes
Source: Chembiochem. 2017 Jul 10;18(16):1604–15. doi: 10.1002/cbic.201700283 (PMC5724660; doi:10.1002/cbic.201700283)
Supplement: Supplementary file 1 — Supplementary [file CBIC-18-1604-s001.pdf]

CHEMBIOCHEM

Supporting Information

Probing Human Telomeric DNA and RNA Topology and Ligand Binding in a Cellular Model by Using Responsive Fluorescent Nucleoside Probes

Sudeshna Manna, Cornelia H. Panse, Vyankat A. Sontakke, Sarangamath Sangamesh, and Seergazhi G. Srivatsan*[a]

cbic_201700283_sm_miscellaneous_information.pdf

Content

Content	Page No
1. Materials	S2
2. Instrumentation	S2
3. Photophysical properties of 5-benzofuran-modified 2′-deoxyuridine (1) and uridine (2)	S2
**Table S1.** Fluorescence properties of nucleoside analogs **1** and **2** in solvents of different polarity and viscosity	S3
**Figure S1.** A working model depicting the location of the probe in RM at low and high w_0 values	S4
**Figure S2.** HPLC chromatograms of PAGE purified fluorescent ONs **5**–**8** at 260 nm	S4
**Figure S3.** MALDI-TOF MS spectrum of modified H-Telo DNA ON **5**	S5
**Table S2.** Extinction coefficient and mass of modified H-Telo DNA and RNA ONs **5**–**8**	S5
**Figure S4**. CD spectra of fluorescently modified H-Telo DNA ONs **5**–**7** and control unmodified H-Telo DNA ON **9** in aqueous buffer	S6
**Figure S5.** CD spectra of fluorescently modified TERRA ON **8** and control unmodified TERRA ON **10** in aqueous buffer	S6
**Figure S6.** UV-thermal melting profile of fluorescent H-Telo DNA ONs **5**–**7** and control unmodified DNA ON **9** in aqueous buffer	S7
**Figure S7.** UV-thermal melting profile of fluorescent TERRA RNA ON **8** and control unmodified TERRA ON **10** in aqueous buffer	S7
**Table S3.** T_m values of modified ONs (**5**–**8**) and control unmodified ONs **9** and **10**	S8
**Figure S8.** Steady-state fluorescence spectra of H-Telo DNA ONs **5**–**8** and their duplexes in aqueous buffer	S8
**Figure S9.** CD spectra of fluorescent H-Telo DNA ONs **5**–**7** and control H-Telo DNA ON **9** in aqueous buffer and AOT RM	S9
**Figure S10.** Steady-state fluorescence spectra and CD spectra of H-Telo DNA **5** in aqueous buffer contnaining different ratios of KCl:NaCl	S9
**Figure S11.** CD spectra of fluorescent TERRA ON **8** and control TERRA ON **10** in aqueous buffer and AOT RM	S10
**Figure S12.** Emission spectra and Curve fit for the binding of PDS to H-Telo RNA ON **8** in aqueous buffer	S10
**Figure S13.** Emission spectra of H-Telo DNA ON **6** and **7** in AOT RM containing NaCl as a function of increasing concentration of PDS	S11
4. References	S11

1. Materials. Dioctyl sodium sulfosuccinate (AOT) and pyridostatin (PDS) were obtained from Sigma-Aldrich. AOT was dried under vacuum for 48 h before use. *n*-heptane (HPLC grade) was purchased from RANKEM, India. *N*-benzoyl-protected dA, *N*,*N*-dimethylformamide-protected dG and *N*-acetyl-protected dC phosphoramidite substrates for solid-phase DNA synthesis were obtained from Proligo Reagents. Solid supports for DNA and RNA synthesis and TBDMS-protected ribonucleoside phosphoramidite substrates were purchased from GLEN Research. All other reagents for solid phase oligonucleotide (ON) synthesis were obtained from either ChemGenes corporation or Sigma Aldrich. ONs **9** and **11** were purchased from Integrated DNA Technologies. Custom ONs were purified by polyacrylamide gel electrophoresis (PAGE) under denaturing conditions and desalted on Sep-Pak Classic C18 cartridges (Waters Corporation). Custom synthesized RNA ON **10** was purchased from Dharmacon RNAi Technologies, PAGE-purified and desalted using Sep-Pak Classic C18 cartridge. Millipore water was autoclaved and used in all biophysical experiments.

2. Instrumentation. Mass measurements were done on an Applied Biosystems 4800 Plus MALDI TOF/TOF analyzer. HPLC analysis of ONs was performed using Agilent Technologies 1260 Infinity HPLC. UV-thermal melting analysis of ONs was performed on a Cary 300Bio UV-Vis spectrophotometer. CD spectra were recorded on a JASCO-J-815 CD spectrometer. Steady-state and time-resolved fluorescence experiments were carried out in a micro fluorescence cuvette (Hellma, path length 1.0 cm) on Fluorolog-3 and TCSPC instrument (Horiba Jobin Yvon, Fluorolog-3), respectively.

3. Photophysical properties of 5-benzofuran-modified 2′-deoxyuridine (1) and uridine (2).[S1,S2] The fluorescence properties of 5-benofuran-modified nucleoside analogs are highly sensitive to polarity and viscosity changes. In a polar solvent like water, the analogs show higher quantum yield with red-shifted emission maximum and longer excited-state lifetime compared to in a nonpolar solvent like dioxane. As the benzofuran and uridine rings are connected by a rotatable aryl-aryl bond, their relative conformation is highly sensitive to the viscosity. In high viscous solvent, due to restricted rotation, the nucleoside analogs show high quantum yield and longer lifetime (Table S1).

Table S1. Fluorescence properties of nucleoside analogs **1** and **2** in solvents of different polarity and viscosity.[S1,S2]

Nucleoside Analog	Solvent	λ_{max} [a]	λ_{em} (nm)	I_{rel} [b]	ϕ	τ_{av} [c] (ns)	r [c]
**1**	water	322	446	1.0	0.190	2.38	0.018
	methanol	322	423	0.8	0.120	0.78	nd
	acetonitrile	322	411	0.3	0.040	0.27	nd
	dioxane	322	406	0.4	0.070	0.33	nd
	ethylene glycol	322	429	2.0	0.399	2.50	0.140
	glycerol	322	427	2.7	0.537	3.56	0.323
**2**	water	322	447	1.0	0.212	2.55	0.016
	methanol	322	423	0.8	0.145	0.94	nd
	acetonitrile	322	410	0.4	0.063	0.33	nd
	dioxane	322	404	0.6	0.099	0.43	nd
	ethylene glycol	322	428	1.9	0.377	3.13	0.125
	glycerol	322	427	2.4	0.528	3.35	0.315

[a]lowest absorption energy maximum is given. [b]Relative emission intensity is given with respect to intensity in water. [c]Standard deviations for τ_{av} (average lifetime) and r (anisotropy) in different solvents are ≤ 0.02 ns and ≤ 0.003 respectively. nd = not determined.

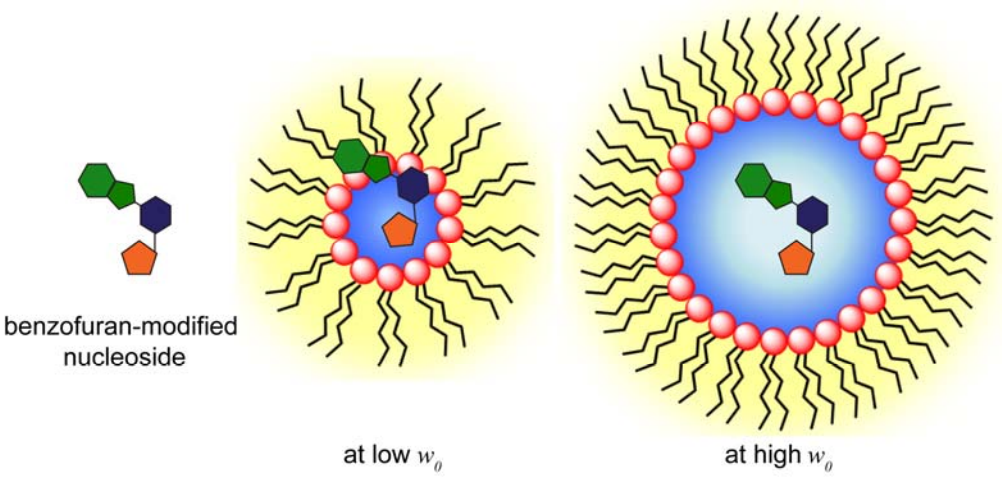

Figure S1. Based on the fluorescence properties of ribonucleoside probe **2** in AOT RM at different w_0 values a working model depicting the location of the probe in RM at low and high w_0 values is given. At low w_0 values (0.6–2.2), the nucleoside is solubilized in a less polar and viscous domain at the interface of polar head groups and structured water associate with the head groups. At higher w_0 values (above 11), well defined water pool appears, and the nucleoside permeates to more polar and less viscous water pool.[S3,S4]

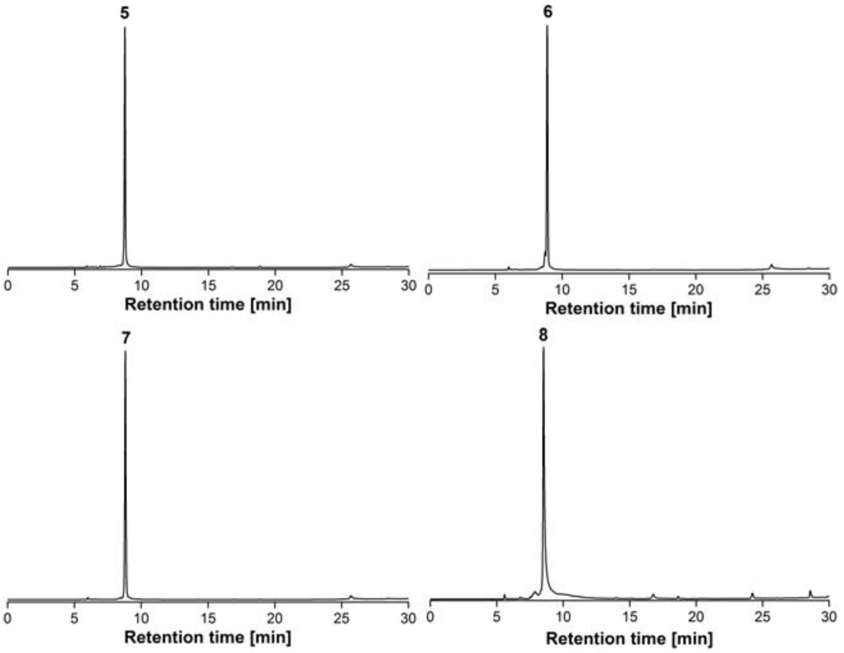

Figure S2. HPLC chromatograms of PAGE purified fluorescent ONs **5–8** at 260 nm. Mobile phase A = 50 mM triethylammonium acetate buffer (pH 7.5), mobile phase B = acetonitrile. Flow rate = 1 mL/min. Gradient = 0–100% B in 30 min. HPLC analysis was performed using Luna C18 column (250 x 4.6 mm, 5 micron).

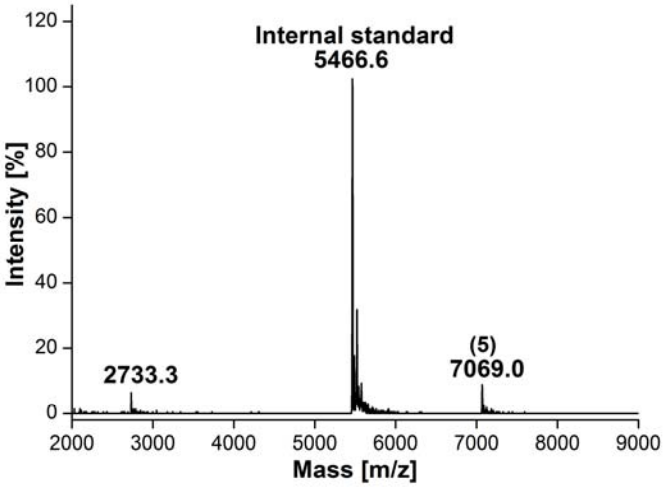

Figure S3. MALDI-TOF MS spectrum of modified H-Telo DNA ON **5** calibrated relative to the +1 and +2 ion of an internal 18-mer DNA ON standard (m/z for +1 and +2 ions are 5466.6 and 2733.3, respectively). Calcd. for **5**: 7068.6 $[M]^+$; found: 7069.0.

Table S2. Extinction coefficient and mass of modified H-Telo DNA and RNA ONs **5**–**8**.

H-Telo DNA ON	$\varepsilon_{260}{}^{a}$ ($M^{-1}cm^{-1}$)	Calculated mass	Observed mass
**5**	232713	7068.6	7069.0
**6**	232713	7068.6	7068.6
**7**	232713	7068.6	7067.9
**8**	263253	7962.8	7962.9

[a] Molar absorption coefficient ε of the modified ONs was determined by using OligoAnalyzer 3.1, which was used for the determination of concentration of modified ONs. The extinction coefficient of nucleoside **1** (ε_{260} = 12613 $M^{-1}cm^{-1}$) and **2** (ε_{260} = 17253 $M^{-1}cm^{-1}$) was used in the place of thymidine and uridine, respectively.

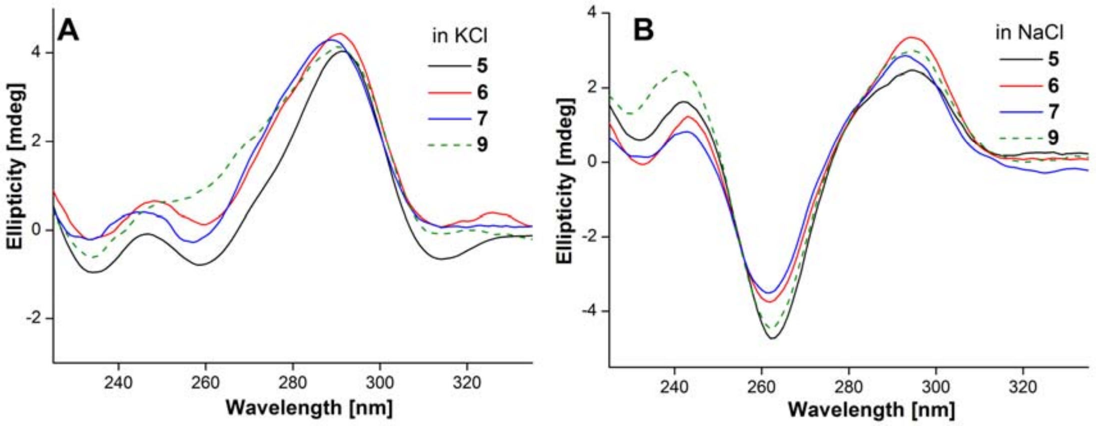

Figure S4. CD spectra of fluorescently modified H-Telo DNA ONs **5**–**7** (8 μM, solid lines) and control unmodified H-Telo DNA ON **9** (8 μM, dashed green lines) in aqueous buffer. (**A**) In Tris-HCl buffer (pH 7.5) containing 50 mM KCl and (**B**) in Tris-HCl buffer (pH 7.5) containing 50 mM NaCl.

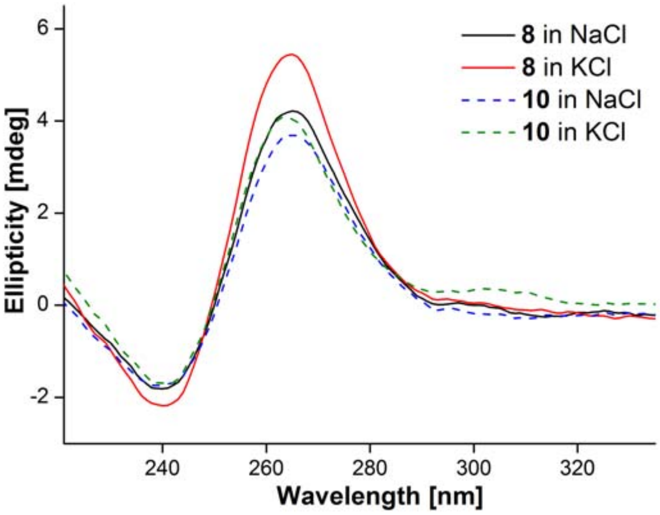

Figure S5. CD spectra of fluorescently modified TERRA ON **8** (8 μM, solid lines) and control unmodified TERRA ON **10** (8 μM, dashed lines) in Tris-HCl buffer (pH 7.5) containing either 50 mM KCl or 50 mM NaCl.

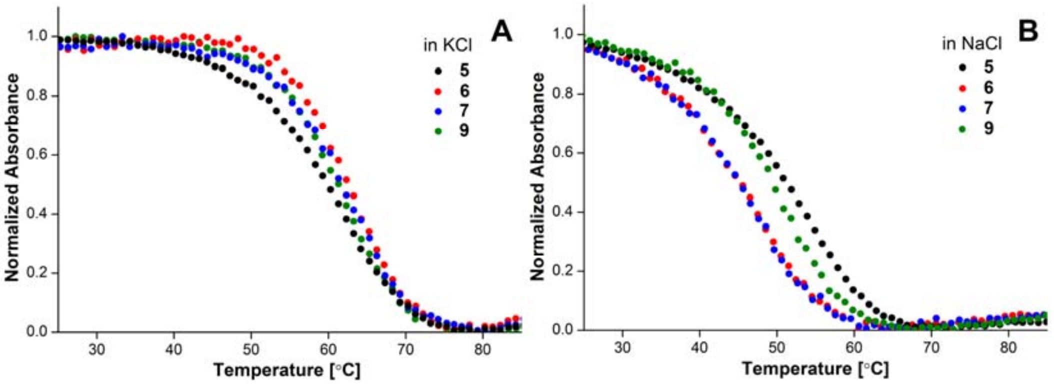

Figure S6. UV-thermal melting profile of fluorescent H-Telo DNA ONs **5**–**7** (1 μM) and control unmodified DNA ON **9** (1 μM) in Tris-HCl buffer (pH 7.5) containing 50 mM KCl (**A**) and 50 mM NaCl (**B**) at 295 nm. For T_m values see Table S3.

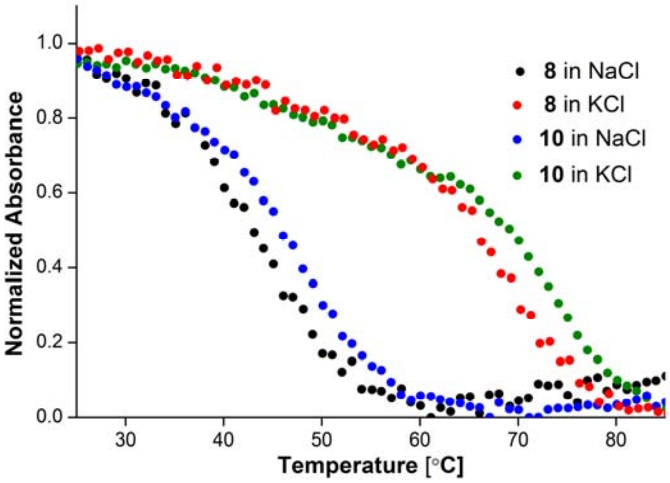

Figure S7. UV-thermal melting profile of fluorescent TERRA RNA ON **8** (1 μM) and control unmodified TERRA ON **10** (1 μM) in Tris-HCl buffer (pH 7.5) containing either 50 mM KCl or 50 mM NaCl at 295 nm. For T_m values see Table S3.

Table S3. T_m values of modified ONs (**5**–**8**) and control unmodified ONs **9** and **10**.

Modified and control unmodified H-Telo DNA and RNA ONs	T_m (°C) in NaCl	T_m (°C) in KCl
H-Telo DNA **5**	53.9 ± 1.1	61.5 ± 0.2
H-Telo DNA **6**	48.0 ± 1.0	63.0 ± 1.0
H-Telo DNA **7**	49.2 ± 0.5	62.8 ± 0.5
Control H-Telo DNA **9**	52.2 ± 0.9	62.0 ± 0.7
TERRA **8**	44.9 ± 0.9	71.1 ± 0.7
control TERRA **10**	46.9 ± 0.1	73.0 ± 0.5

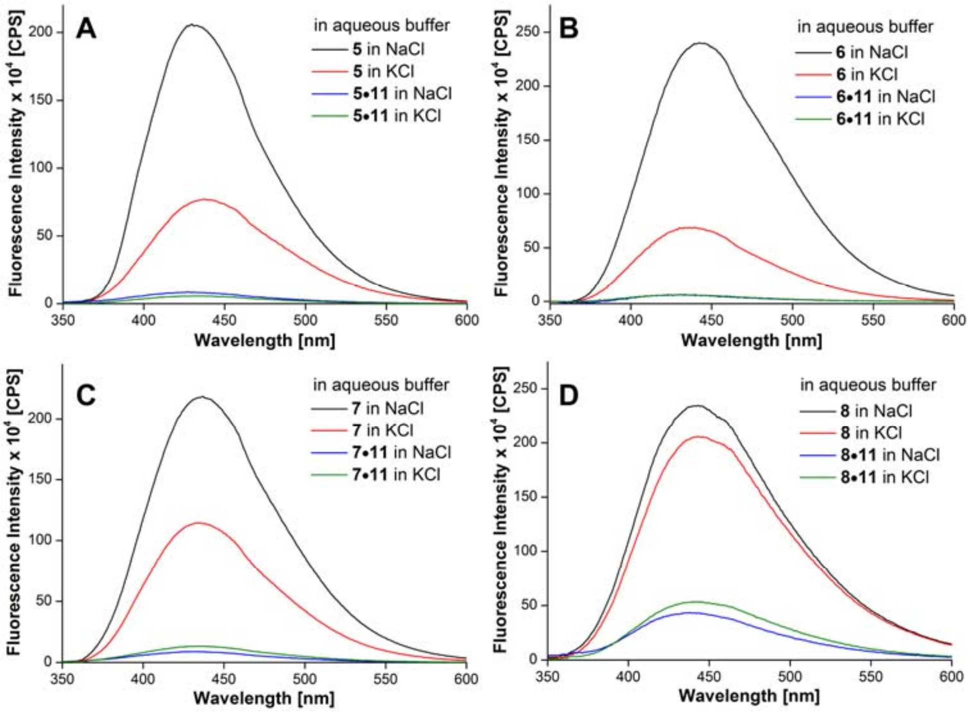

Figure S8. Steady-state fluorescence spectra of (**A**) H-Telo DNA ON **5** and corresponding duplex **5•11**, (**B**) H-Telo DNA ON **6** and corresponding duplex **6•11**, (**C**) H-Telo DNA ON **7** and corresponding duplex **7•11** and (D) TERRA ON **8** and corresponding duplex **8•11** in Tris-HCl buffer (pH 7.5) containing 50 mM NaCl or 50 mM KCl. DNA ON samples (1 µM) were excited at 322 nm with an excitation and emission slit width of 3 nm and 4 nm, respectively. RNA ON samples (0.26 µM) were excited at 322 nm with an excitation and emission slit width of 6 nm and 8 nm, respectively.

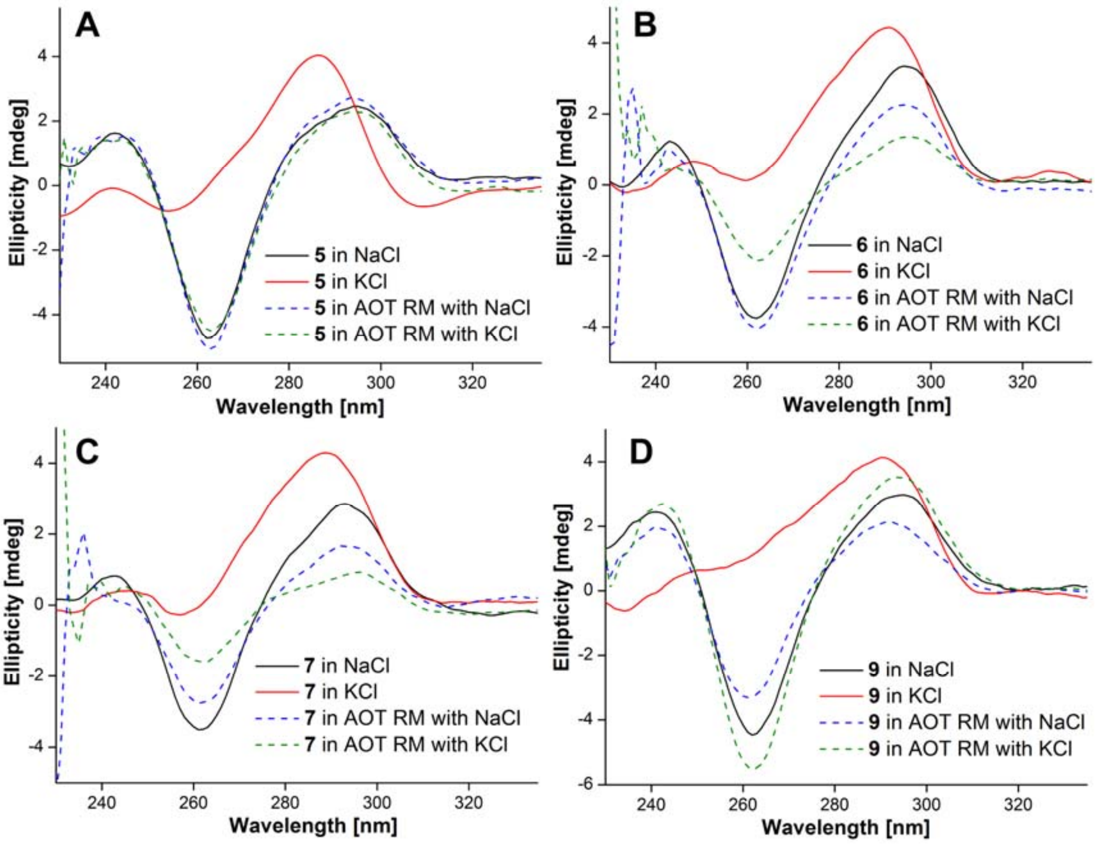

Figure S9. CD spectra of fluorescent H-Telo DNA ONs **5**–**7** and control H-Telo DNA ON **9** in aqueous buffer (solid lines) and AOT RM (200 mM in *n*-heptane, dashed lines).

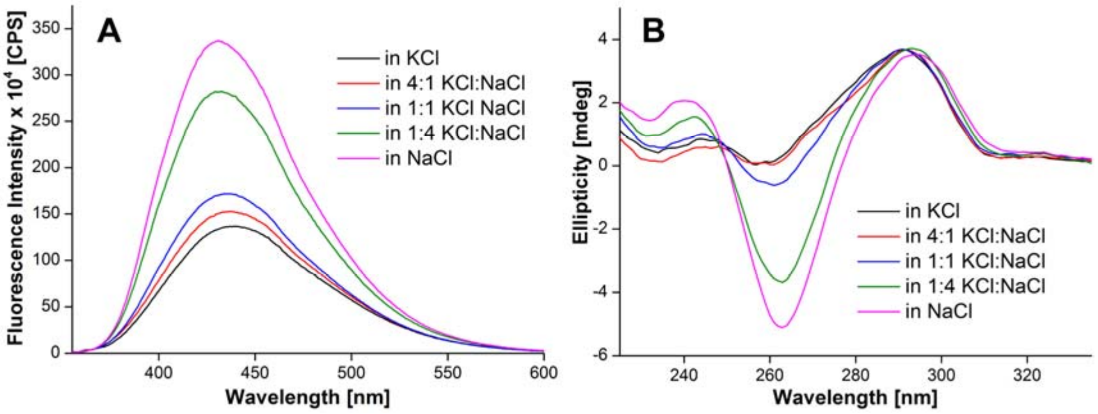

Figure S10. (**A**) Steady-state fluorescence spectra (0.26 µM) and (**B**) CD spectra (8 µM) of H-Telo DNA **5** in aqueous buffer contnaining different ratios of KCl:NaCl. Total NaCl and KCl concentration was maintiained at 50 mM.

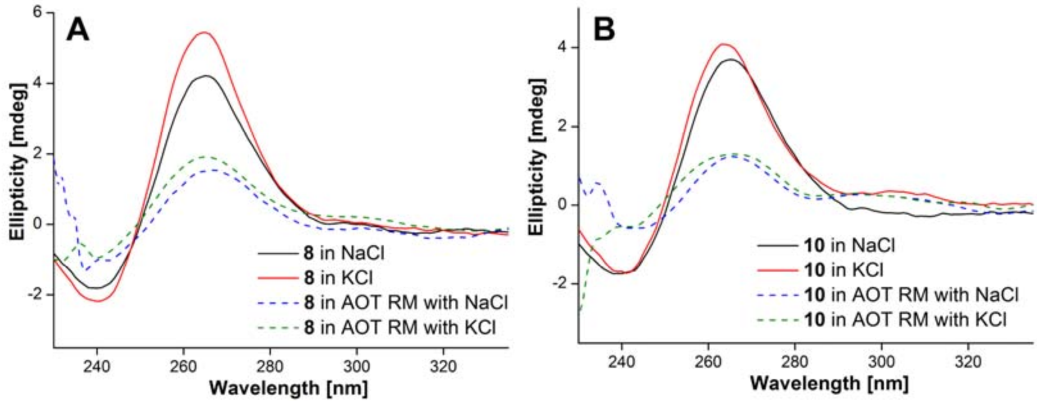

Figure S11. CD spectra of fluorescent TERRA ON **8** and control TERRA ON **10** in aqueous buffer (solid lines) and AOT RM (dashed lines).

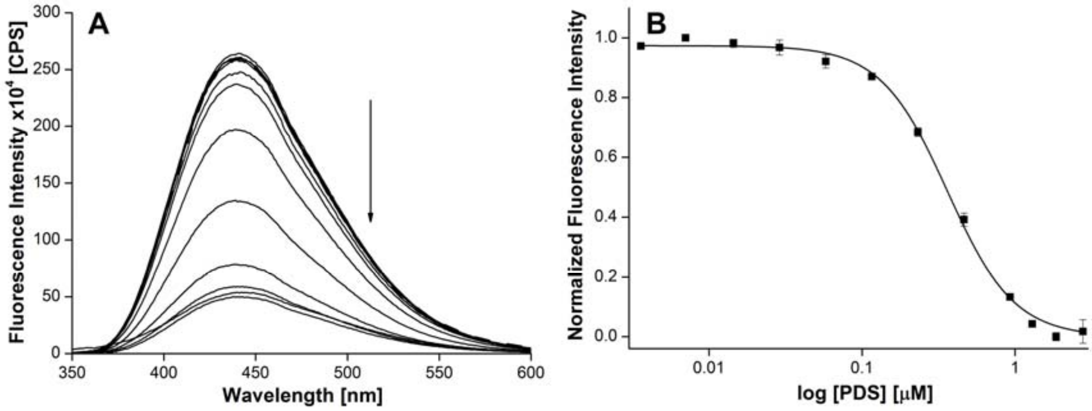

Figure S12. (**A**) Emission spectra of H-Telo RNA ON **8** (0.26 μM) in aqueous buffer containing NaCl (50 mM) as a function of increasing concentration of PDS. Dashed line represents fluorescence spectrum of **8** in the absence of PDS. The samples were excited at 322 nm with an excitation and emission slit width of 6 nm and 8 nm, respectively. (**B**) Curve fit for the binding of PDS to H-Telo RNA ON **8** in aqueous buffer. Normalized fluorescence intensity at 441 nm is plotted against log [PDS].

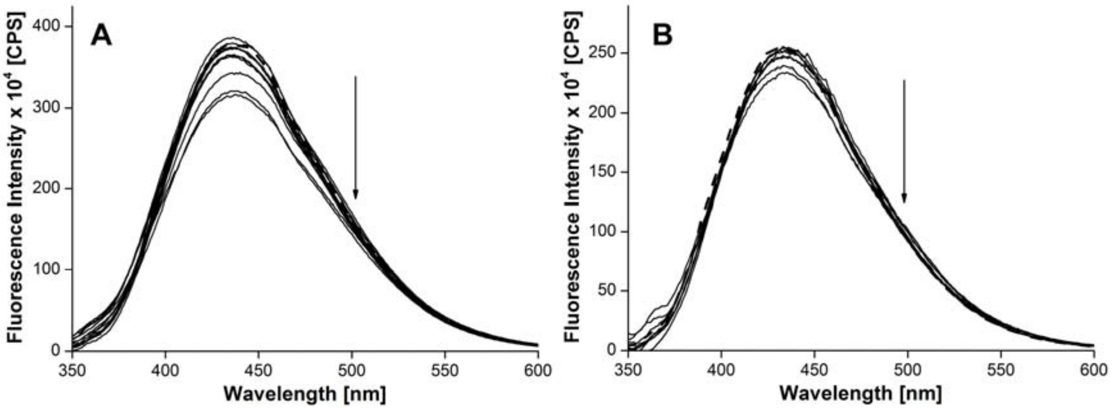

Figure S13. Emission spectra of H-Telo DNA ON (**A**) **6** (0.28 μM) and (**B**) **7** (0.28 μM) in AOT RM containing NaCl (50 mM) as a function of increasing concentration of PDS. Dashed line represents fluorescence spectrum of ON in the absence of PDS. The samples were excited at 322 nm with an excitation and emission slit width of 4 nm and 6 nm, respectively.

11. References

[S1] A. A. Tanpure and S. G. Srivatsan, *ChemBioChem* **2012**, *13*, 2392–2399.

[S2] A. A. Tanpure, S. G. Srivatsan, *Chem. Eur. J.* **2011**, *17*, 12820–12827.

[S3] K. K. Karukstis, A. A. Frazier, D. S. Martula, J. A. Whiles, *J. Phys. Chem.* **1996**, *100*, 11133–11138.

[S4] B. Sengupta, J. Guharay, P. K. Sengupta, *Spectrochim. Acta A* **2000**, *56*, 1433–1441.
